# Discrimination against childbearing Romani women in maternity care in Europe: a mixed-methods systematic review

**DOI:** 10.1186/s12978-016-0263-4

**Published:** 2017-01-05

**Authors:** Helen L. Watson, Soo Downe

**Affiliations:** 1 Sheffield Teaching Hospitals NHS Foundation Trust, Jessop Wing, Tree Root Walk, Sheffield, S10 2SF UK; 2 Research in Childbirth and Health (ReaCH) group, University of Central Lancashire, Preston, PR3 2LE UK

**Keywords:** Discrimination, Human rights, Maternity care, Mistreatment, Roma, Prejudice, Health mediation

## Abstract

**Background:**

Freedom from discrimination is one of the key principles in a human rights-based approach to maternal and newborn health.

**Objective:**

To review the published evidence on discrimination against Romani women in maternity care in Europe, and on interventions to address this.

**Search strategy:**

A systematic search of eight electronic databases was undertaken in 2015 using the terms “Roma” and “maternity care”. A broad search for grey literature included the websites of relevant agencies.

**Data extraction and synthesis:**

Standardised data extraction tables were utilised, quality was formally assessed and a line of argument synthesis was developed and tested against the data from the grey literature.

**Results:**

Nine hundred papers were identified; three qualitative studies and seven sources of grey literature met the review criteria. These revealed that many Romani women encounter barriers to accessing maternity care. Even when they are able to access care, they can experience discriminatory mistreatment on the basis of their ethnicity, economic status, place of residence or language. The grey literature revealed some health professionals held underlying negative beliefs about Romani women. There were no published research studies examining the effectiveness of interventions to address discrimination against Romani women and their infants in Europe. The Roma Health Mediation Programme is a promising intervention identified in the grey literature.

**Conclusions:**

There is evidence of discrimination against Romani women in maternity care in Europe. Interventions to address discrimination against childbearing Romani women and underlying health provider prejudice are urgently needed, alongside analysis of factors predicting the success or failure of such initiatives.

**Electronic supplementary material:**

The online version of this article (doi:10.1186/s12978-016-0263-4) contains supplementary material, which is available to authorized users.

## Plain english summary

All childbearing women in Europe are entitled to be free from experiencing discrimination in maternity health care. The Roma are the largest and most marginalised ethnic minority group in Europe and experience discrimination in many areas of life. This review aimed to investigate published evidence about Romani women’s experiences of discrimination in maternity care in Europe and any interventions to address this. The review identified 900 papers, and after eligibility and quality assessment, three published qualitative studies and seven sources of non-research literature were taken forward for analysis. These revealed that many Romani women encounter barriers to accessing maternity care. Even when they are able to access care, they can experience mistreatment that is discriminatory on the basis of their ethnicity, economic status, place of residence or language. The non-research literature revealed some health professionals held underlying negative beliefs about Romani women. There were no published research studies examining the effectiveness of interventions to address discrimination against Romani women and their infants in Europe. The Roma Health Mediation Programme is a promising intervention identified in the non-research literature. The development of further interventions to address discrimination against childbearing Romani women and underlying health provider prejudice are urgently needed, alongside analysis of factors predicting the success or failure of such initiatives.

## Introduction

There is now wide acceptance of the relationship between human rights and maternal and infant health and wellbeing [1]. A human rights-based approach to health is a key feature of emerging global health policy within the post-2015 agenda, including the Global Strategy for Women’s and Children’s Health and the Sustainable Development Goals [2, 3]. International efforts to improve access to and quality of maternal and newborn care are often hindered by the failure to eradicate discrimination in both policy development and the provision of services [4]. There is a scarcity of studies within the academic literature that examine the implementation or impact of a human rights-based approach to maternal and infant health care [5]. This paper presents the findings of a systematic review of existing literature in this area, focused on the experiences of Romani women.

## Background

Human rights are basic values that are essential to human dignity [6], and concern the empowerment and entitlement of people with respect to certain aspects of their lives, including their sexual and reproductive health [7]. Discrimination is prohibited in the Universal Declaration of Human Rights and in other treaties in relation to the exercise and enjoyment of covenant rights. Within human rights law discrimination is defined as;

Any distinction, exclusion, restriction or preference or other differential treatment that is directly or indirectly based on the prohibited grounds of discrimination and which has the intention or effect of nullifying or impairing the recognition, enjoyment or exercise, on an equal footing, of Covenant rights ([8], p. 3).

Discrimination may be against an individual belonging to, perceived to belong to or associated with a group with characteristics defined within the prohibited grounds of discrimination (See Table 1). It is motivated by socially derived beliefs and ideologies about specific groups in society that justify patterns of behaviour to enact dominance or oppression and to obtain power or privilege [9–11].

**Table 1:** Prohibited grounds of discrimination

Race and colour	
Sex	
Language	
Religion	
Political or other opinion	
National or social origin	
Property	
Birth	
Disability	
Age	
Nationality	
Martial and family status	
Sexual orientation and gender identity	
Health status	
Place of residence	
Economic and social situation [8]	

### Discrimination in maternity care

The human rights of childbearing women incorporate the right to freedom from discrimination and the principle of non-discrimination in the exercise and enjoyment of numerous other covenant rights, not least the right to the highest attainable standard of health. Discrimination against women on the basis of gender or other grounds is implicated in preventable maternal mortality and morbidity [12, 13], and particularly affects women living in low income countries, rural areas, in poverty or belonging to ethnic minority groups [14]. A human rights-based approach to address maternal mortality and morbidity identifies that targeted measures are required to ensure the rights of marginalised groups [15].

In order to ensure women’s sexual and reproductive health rights, health facilities, goods and services should meet standards of; availability, accessibility, acceptability and quality [7]. Non-discrimination is a vital dimension of accessibility and States are required to ensure that measures are taken to eliminate barriers that women face in gaining access to healthcare [7, 16]. Non-discrimination is also a component of respectful maternity care, which is a critical dimension of quality and acceptability standards [7], and States should ensure that health services respect women’s dignity and are sensitive to their needs and perspectives [16].

There is a growing body of global research that demonstrates many childbearing women experience discrimination in maternity care on the basis of their ethnicity, race, religion, socioeconomic status, age, marital status, medical conditions or sexual orientation [17, 18]. This discrimination results in differential treatment that influences the quality of care the women receive, breaches of confidentiality, humiliation, women feeling alienated from their caregivers and women choosing to avoid the health facility for their next birth [17]. Data from wider studies in other areas of healthcare provision confirm that discrimination in the healthcare setting results in delays in seeking care, reluctance to follow the advice of medical practitioners and lower use of preventative services [19–21]. All of these, in the context of maternity care, may result in reduced access or uptake of services and poor maternal and infant outcomes.

### The Roma

The Roma are considered to be the largest and most marginalised ethnic minority in Europe [22, 23], and although precise figures are unknown, are estimated to number between ten and twelve million people [24, 25]. Roma are found in most European countries with the majority residing in Central and Eastern European countries [22] and less than 20% reporting to be nomadic [26].

The United Nations have recently clarified:

The term “Roma” refers to heterogeneous groups, the members of which live in various countries under different social, economic, cultural and other conditions. The term Roma thus does not denote a specific group but rather refers to the multifaceted Roma universe, which is comprised of groups and subgroups that overlap but are united by common historical roots, linguistic communalities and a shared experience of discrimination in relation to majority groups. “Roma” is therefore a multidimensional term that corresponds to the multiple and fluid nature of Roma identity. ([27], p. 3)

Throughout their history the Roma have been consistently subjected to harassment and persecution. The repression of Roma in Europe reached its peak in what is termed, in the language of the Roma, “porrajmos” translated as “the devouring”; the extermination of between 220,000 and 1,500,000 Roma that began in 1940 and continued during World War II as part of the holocaust [28, 29].

Several United Nations bodies have identified that ongoing discrimination against the Roma results in racial violence and impacts on their rights to; education, health, housing, employment, political participation, access to citizenship and justice, and on the minority rights of existence, protection and promotion of collective identity and participation in public life [1, 27, 30, 31].

There are limited data and few studies that consider the health of Romani women within Europe [26]. The data that are available indicate that Romani women are more disadvantaged and suffer worse health than Roma men and the non-Roma [26, 32]. They have less access to family planning supplies, higher birth rates, higher numbers of teenage pregnancies, higher rates of illegal or unsafe abortions, and lower uptake of cervical screening [33, 34]. They have higher rates of poor infant outcomes including low birthweight and preterm birth, and face multiple barriers to accessing healthcare services [26]. In 2011 the European Council adopted the European Union (EU) Framework for National Roma Integration Strategies, which links social and economic inclusion with the elimination of discrimination [35]. All EU Member States have since adopted National Roma Integration Strategies, or integrated national policies based on this framework [35]. In the area of health, the strategy specifies that States should ensure Romani women have access to quality healthcare in line with the principle of non-discrimination [36].

### The review

Based on the background above a mixed-methods systematic review of existing literature was designed to fulfil the following aims;

1. To establish the current evidence base in terms of discrimination against Romani women in maternity care in Europe.
2. To assess the nature, effectiveness of, and underlying mechanisms of interventions designed to address discrimination against Romani women and their infants within the design and/or provision of maternity care in Europe.

### Design

A mixed-methods systematic review was undertaken, informed by philosophical pragmatism with a complementary axiological framework informed by the transformative paradigm [37, 38]. This paradigm is concerned with social justice, societal power differences and their ethical implications including discrimination and oppression, and aims to increase the visibility of members of communities who have been marginalised within society [39].

An “a priori” protocol was developed using guidance from Hayvaet et al. [40], The Centre for Reviews and Dissemination [41], The Cochrane Handbook for Systematic Reviews of Interventions [42], The Joanna Briggs Institute [43], and the segregated model of data synthesis proposed by Sandelowski et al. [44]. The terms within the research questions were defined, and inclusion and exclusion criteria developed using the PICOS acronym; population, intervention, comparators, outcomes and study design [41, 42].

It was intended that both types of secondary data, qualitative and quantitative, would be given equal weighting in the analysis, would be collected simultaneously and integrated at the synthesis phase.

### Definition of terms

- Romani women - women identified or self-identifying as Romani
- Maternity care - maternal and infant health goods, services and facilities provided during pregnancy, birth, the postnatal period, and through to the early weeks of life
- Europe – countries belonging to the Council of Europe (Table 2)

**Table 2:** Countries belonging to the Council of Europe

Albania Andorra Armenia Austria Azerbaijan	Belgium Bosnia and Herzegovina Bulgaria,	Croatia, Cyprus, Czech Republic,	Denmark
Estonia	Finland France	Georgia Germany Greece	Hungary
Iceland Ireland Italy	Latvia Liechtenstein Lithuania Luxemborg	Malta Moldova Monaco Montenegro	Netherland Norway
Poland Portugal	Romania Russian Federation	San Marino Serbia Slovak Republic Slovenia Spain Sweden Switzerland	“The former Yusgoslav Republic of Macedonia” Turkey
Ukraine			
United Kingdom
- Discrimination in maternity care:

*Any differential treatment that is directly or indirectly based on the prohibited grounds of discrimination and which has the intention or effect of nullifying or impairing the exercise, on an equal footing, of childbearing women’s right to maternal and infant health facilities, goods and services that are accessible, acceptable, available and of good quality. This includes when the design or provision of maternity care appears neutral at face value, but has a disproportionately negative impact on women and their infants on the basis of prohibited grounds.*

### Inclusion and exclusion criteria

These are given in Table 3.

**Table 3:** Inclusion and exclusion criteria

Population	Romani women or their infants living in countries that are members of the Council of Europe
	Maternity healthcare staff working with Romani women in countries that are members of the Council of Europe
Intervention (Question 2 only)	Aims to address discrimination in maternity care
	Occurs during pregnancy or the postnatal period up to 42 days after birth
	Addresses the design or provision of maternal and newborn care
	Not interventions with the newborn in the absence of involving the mother
	Not interventions limited to reproductive technologies or termination services
Control (Question 2 only)	Study includes a control group who did not receive the intervention or program
Outcomes	The accessibility or availability or acceptability or quality of maternal or infant health goods, facilities or services
	Data from health care workers about their experience of caring for childbearing Romani women or their attitudes/beliefs about childbearing Romani women and their infants
Study Type	Any peer reviewed quantitative, quantitative or mixed-methods primary research studies or systematic reviews of these studies.
	Only studies with full text
	Grey literature
Language	Available in English
Date	No date limits

### Search strategy

A broad search strategy was used which included terms for “maternity care” and “Roma”. The search strategy used keywords rather than MeSH terms so that it was easily transferable between databases, and covered synonyms, related terms, and spelling variations, and used wildcard and truncation functions to ensure it was as comprehensive as possible [42]. The development of the search terms was an iterative process, and the final combination of terms using Boolean operators is detailed in Additional file 1. This search was undertaken in May 2015 in Medline, EMBASE, Maternal and Infant Care via Ovid SP, AMED, CINAHL, Academic Search Complete, PsychINFO and Wilson Social Science Abstracts via EBSCOhost EJS and PROSPERO.

The search for grey literature was undertaken separately by reference tracking from relevant articles in the initial search, searching the websites of relevant agencies including World Health Organisation, Council of Europe, United Nations Population Fund (UNFPA), Open Society Foundation, Amnesty International, and broad internet searching using Google search engine.

### Data extraction and quality appraisal

Data were extracted using a standardised electronic form that was refined iteratively. The Critical Appraisal Skills Programme (CASP) checklists were selected for the appraisal of cohort studies, case–control studies and randomised control studies [45] Greenhalgh et al’s [46] tool was chosen for mixed-method studies appraisal, the Assessing the Methodological Quality of Systematic Reviews (AMSTAR) tool for the appraisal of systematic reviews [47], and the tool developed by Walsh and Downe [48] for appraising qualitative studies.

The quality of the studies was grading by adopting the system first published by Downe, Simpson and Trafford [49], and as used by Shaw, Downe and Kingdon [50]. This grading system uses a four category coding, from A+ (highest quality) to D- (very poor quality). Quantitative papers were to be graded against the criteria of internal validity, reliability, replicability and generalisability [51], and qualitative papers against criteria identified by Lincoln and Guba [52]; credibility, transferability, dependability and confirmability (See Table 4). Papers graded C+ or below were excluded.

**Table 4:** Grading criteria for quality of qualitative studies

Qualitative papers	
Graded against the criteria of internal validity, reliability, replicability and generalisability [51].	
A	No, or few flaws. The study’s internal validity, reliability, replicability and generalisability are high.
B	Some flaws, unlikely to affect the internal validity, reliability, replicability and generalisability of the study.
C	Some flaws that may affect internal validity, reliability, replicability and generalisability of the study.
D	Significant flaws that are very likely to affect the internal validity, reliability, replicability and generalisability of the study.
Quantitative papers	
Graded against criteria identified by Lincoln and Guba [52]; credibility, transferability, dependability and confirmability.	
A	No, or few flaws. The study’s credibility, transferability, dependability and/or confirmability are high.
B	Some flaws, unlikely to affect the credibility, transferability, dependability and/or confirmability of the study.
C	Some flaws that may affect the credibility, transferability, dependability and/or confirmability of the study.
D	Significant flaws that are very likely to affect the credibility, transferability, dependability and/or confirmability of the study.

### Data synthesis

It was intended that the data would be synthesised using the segregated method [44], in which quantitative and qualitative data are synthesised separately, meta-analytically and through meta-synthesis respectively, and then integrated together in a line of argument synthesis

The intention was to synthesise the quantitative data meta-analytically unless too heterogeneous, in which case a narrative method would be undertaken. The chosen meta-synthesis method was based on the methods of Finlayson and Downe [53] and Walsh and Downe [54], both of which were developed from meta-ethnography [55]. The grey literature was synthesised separately using a simple narrative method. It was intended that comparison with the grey literature would allow the line of argument to be tested and refined. The intention was to ensure the integrity of the original research within the synthesis and to incorporate quotes from the original respondents in the primary sources to provide some internal validity to the synthesis [56].

Confidence of the review qualitative findings were assessed using the Confidence in the Evidence from Reviews of Qualitative Research (CERQual) approach [57]. This method involved an assessment of confidence in the second order themes using the four CERQual domains; methodological limitations, relevance, adequacy of data and coherence [57]. Each finding was scored on each domain (very low to high) and then an overall score determined.

## Results

### Search outcome

**Fig. 1:**
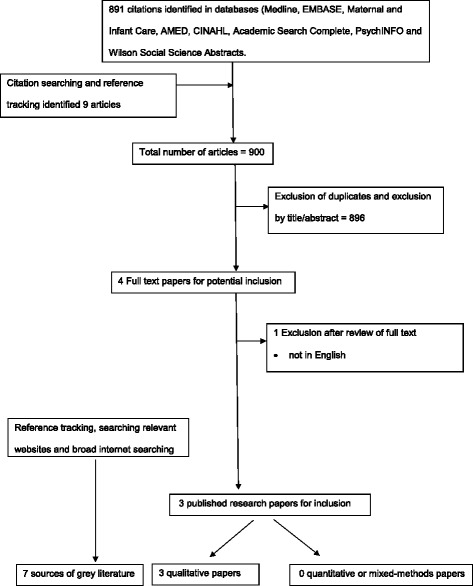
Flow diagram of study selection

Figure 1 gives the results of the search. Nine hundred articles were identified, and after the removal of duplicates, and screening of the titles and abstracts against the inclusion criteria, four peer reviewed papers met the inclusion criteria and were taken forward and read in full. After final screening, one further paper was excluded on the basis of language (See Additional file 2). The characteristics of the included studies can be seen in Table 5.

**Table 5:** Characteristics of the included published research studies

Author and countries	Focus	Design and methods	Sampling strategy	Sample characteristics	Analytic strategy	Quality score	Funding
Janevic et al., [58] Serbia and Macedonia	Discrimination and access to prenatal and maternity care amongst Romani women	Community-based participatory research. 8 focus groups In-depth structured interviews	Purposive sampling of Romani women. Snowball sampling of gynaecologists	71 Romani women who had given birth in past year, age 14–44. 8 Gynaecologists	Constant comparison method	B	Not specified
Columbini et al. [59] Albania, Bulgaria, Macedonia	To explore access of Roma in South-Eastern Europe to sexual and reproductive health services	Focus group discussions	Purposive sampling	58 male and female Romani participants	Thematic analysis using AtlasTi	B-	UNFPA, and European Observatory on Health Systems and Policies
Condon and Salmon [60] South-west England	To explore mothers and grandmothers’ views on feeding in the first year of life, including the support provided by health professionals.	1-1 interviews	Not specified	22 women, of whom 11 were Romani. 2 were grandmothers and 9 mothers.	Coding using NVivo 9 and development of themes	B-	University of the West of England as part of the SPUR Early Career Researcher funding stream.

No quantitative or mixed-methods studies met the review criteria. Three qualitative studies were included [58–60]. The studies incorporated data from Serbia, Macedonia, Albania, Bulgaria and England. They ranged in publication date from 2011–2014 and scored acceptable quality ratings of B or B-.

Seven relevant sources of grey literature were identified [36, 61–66]. Their characteristics can be seen in Table 6. Six of these sources included qualitative data from study reports pertaining to Romani women’s experiences of maternity care or access to maternity care in Europe [36, 61–65]. One source was a section of a PhD thesis that analysed the impact of an intervention with childbearing Romani women [66]. The sources ranged in publication date from 2001 to 2014 and incorporated data from 25 European countries, although the majority of the data were from Central and Eastern European regions.

**Table 6:** Characteristics of included grey literature

Author	Aim	Source type	Setting	Study method	Participants	Funding
Pohjolainen [36]	To outline barriers and issues experienced by Romani women in relation to maternal health, and issues raised by practitioners in relation to the provision of inclusive maternal health services for Roma.	Parvee Point Report	Ireland	Findings from the Roma maternal and child health seminar, including speakers, focus group discussions among seminar participants, and interviews with practitioners and members of Roma communities.	Romani women Health professionals Roma Health Seminar participants	Health Service Executive, Ireland.
European Roma Rights Centre [61]	To document discriminatory practices and other forms of human rights abuse against Roma in the provision of health care as well as exclusion from access to health care.	Report for European Roma Rights Centre	Bosnia and Herzegovina, Czech Republic, Croatia, Greece, France, Italy, Kosovo, Romania, Serbia, Slovakia, Slovenia, Bulgaria, Hungary and Spain.	Interviews in Bulgaria Hungary and Spain, research with partner organisations and information from ERRC legal databases	Romani men and women aged 18–70 Physicians treating Roma patients. Number not specified.	Open Society Institute
Iszak [62]	To document practices of discrimination against Romani women within the health care sector in Hungary.	Report published by European Roma Rights Centre	Szabolcs-Szatmár Bereg, Hajdú-Bihar and Borsod-Abaúj-Zemplén counties in Hungary	Interviews	131 Romani women Physicians, number not specified.	Not specified
Centre for Reproductive Rights [63]	To document suspected cases of coerced sterilizations against Romani women who accessed reproductive health services in Slovakia.	Report by Center for Reproductive Rights and Poradna	40 Romani settlements in Eastern Slovakia	Individual interviews and group interviews	230 Romani women 25 doctors, 7 hospital administrators, 6 nurses	Not specified
European Monitoring Centre on Racism and Xenophobia [64]	To investigate the situation of Romani women accessing healthcare.	Report to Council of Europe	Bulgaria, Finland, France, UK. Greece, Hungary, Ireland, Lithuania, Moldova, The Netherlands, Poland, Romania, Serbia and Montenegro, Slovakia and Spain.	Country visits and individual interviews and questionnaires.	Romani women, representatives of governments and NGOs and health workers. Numbers not specified.	UK Government and European Union’s European Monitoring Centre on Racism and Xenophobia
Zoon [65]	To document the ways in which the Romanian, Bulgarian, and Macedonian governments and their representatives discriminate against the Roma in the provision of social protection benefits, health care, and housing.	Open Society Foundation report	Romania, Bulgaria and Macedonia	Interviews	Government officials, legislators, social workers, Romani activists, health workers and Romani residents. Numbers not specified.	Open Society Institute
Benjenariu, & Mitrut [66]	To analyse a large-scale public health program targeting Roma minority, the Roma Health Mediation programme, and its impact on prenatal care and child health	Section of PhD thesis published by University of Gothenburg	Romania	Quantitative analysis of the 2000–2008 Vital Statistics Natality (VSN), Vital Statistics Mortality (VSM) files, and data from the Roma Health Mediator registry and the Roma Inclusion Barometer 2006.	Romani women and their infants born between 2000–2008 (10,885 – 13,685 observations)	Not specified

#### Qualitative findings

The findings from the peer-reviewed papers were compared and grouped into first and second order themes as shown in Table 7. A search was made for any disconfirming or unexplained data and an assessment of each second order theme was undertaken to determine the CERQual score as shown in Table 8. Two second order themes achieved an overall medium CERQual score, as although the findings were well grounded in the data, there were only a very small number of primary studies which represented a limited number of geographical contexts across Europe and the quality score of the studies were medium. A third second order theme achieved an overall low CERQual score as there were very few primary studies containing thin data from a small number of participants and no convincing explanation for contrasting data.

**Table 7:** Qualitative data themes

Second order themes	First order themes
Mistreatment within maternity care	Poor communication
	Being abandoned
	Physical and verbal abuse
	Refused care
	Made to wait until other non-Roma patients seen to
	Negative attitudes of doctors
Barriers to accessing maternity care	Lack of perceived need for care
	Lack of awareness of right to care
	Geographical barriers and transport barriers
	Denial of treatment
	Language barriers
	Financial barriers
	Patriarchal culture
Making things better	Connection with the health centre improving experience
	Knowledge of rights to overcome barriers
	Presence of Romani health workers improving quality of care

**Table 8:** CERQual scores

Themes	Evidence (Study Code)	CERQual score and comments				
		Methodological limitations	Relevance	Adequacy of data	Cohrerence	Overall score
Mistreatment within maternity care	58, 59, 60	Medium	Medium	Low	High	Medium
		Studies with quality scores of B to B-	Studies represented limited number of geographical contexts across Europe	Small number of primary studies (3), although thick data available.	Well grounded in the data	
Barriers to accessing maternity care	58, 59, 60	Medium	Medium	Low	High	Medium
		Studies with quality scores of B to B-	Studies represented limited number of geographical contexts across Europe	Small number of primary studies (3) although thick data available.	Well grounded in the data	
Making things better	58, 59	Medium	Medium	Very low	Low	Low
		Studies with quality scores of B to B-	Studies represented limited number of geographical contexts across Europe	Only 2 primary studies and very thin data from small number of participants	No convincing explanation for contrasting data	

### Mistreatment within maternity care

The Romani women reported a variety of poor experiences that constituted mistreatment within maternity care, including poor communication [58–60], being abandoned [58], physical and verbal abuse [58, 59], being refused care [59] and being made to wait until the non-Roma women had been attended to [59].

The Romani women reported that health care workers, particularly doctors, communicated with them poorly and rarely explained anything about procedures or problems;

“*They are not interested and always tell us that everything is fine, even when it isn’t, and all that just because we are Roma.” (*[59], p.530)

Some women described being abandoned by the medical staff when they were inpatients at the maternity facilities;

*“After the delivery… they placed me in a separate room alone and nobody came to ask me how I feel, the entire night I bleed till 7 am. I could die.” (*[58], p.4)

The women reported they experienced rough treatment particularly during the delivery of their baby [58] and verbal abuse and racial slurs [58, 59];

*“My doctor…she only yells and shouts. They say that she hates Roma” (*[58], p.4)

*“When I gave birth to my sixth child, the midwife told me: ‘Gypsy job! Only Gypsies have so many children!’ It was offensive, I was not happy with this. (*[59], p.530)

Some Romani women reported that medical professionals were unwilling to treat them in medical facilities [58, 59];

*“There are cases in which doctors do not want to examine us. Maybe because we are ‘black’,‘dark’” (*[59], p.530)

The women reported that the medical staff prioritised non-Roma women above them and made them wait unfairly;

*“No matter if we are first in line, we are always checked-up last. The Macedonians and Albanians, they always have priority…all that just because we are Roma.” (*[59], p.530)

### Barriers to accessing care

The Romani women described barriers to accessing care that included; a lack of awareness of their right to care [58], a lack of perceived need for care [58], geographical and transport barriers [58, 59], being denied treatment [58, 59], language barriers [60], and financial barriers [58, 59].

Many women were unaware of their right to maternity care. In one study they questioned the focus group moderators about their rights to social assistance during the research process [58]. Other Romani women did not perceive any need to visit a doctor during pregnancy;

*“I didn’t go to the gynecologist during my pregnancy. Why should I go to the doctor? I knew that I was pregnant. I went to the doctor when I felt my contractions.” (*[58], p.7)

The women reported a lack of local maternal health service provision within their settlements [58] and transport barriers to accessing services including lack of direct bus services [58], the costs of transport [58, 59], and particular difficulties for those living further from the cities [59].

*“It would be much easier, if we have a doctor here, so that we wouldn’t have to roam the road.” (*[59], p.530)

The women identified that they were denied access to healthcare by doctors refusing to register them for primary care services [58], by medical professionals unwilling to treat them in medical facilities [58, 59] or by emergency services who were unwilling to attend when required in Roma settlements [58];

*“But if you call an ambulance here you might die” (*[58], p.6)

Some women reported that health professionals did not provide health information in a language they could understand;

*“We received leaflets in English, about how to breast feed and what to expect when you’re a Mum, but we don’t actually know how to read in English.” (*[60], p.789)

The women described that their lack of finances, inability to give informal payments or bribes and their lack of health insurance or access to private healthcare impacted on their access to care and to the quality of care they received [58, 59]. Their inability to give informal payments or bribes resulted in poorer care and neglect by the health professionals [58, 59] and resulted in health professionals refusing to allow them to have the support of a family member present with them in the maternity unit [58];

*“They asked me to pay 11,000 MKD [equivalent to USD 252]; I didn’t have that kind of money, but they weren’t interested, so I had to give birth to my child at home. The childbirth lasted for two days and I fell unconscious several times.” (*[59], p.528)

*“They looked for money from me, they didn’t want to deliver my baby until my mother-in-law gave them money and then everything was different.” (*[58], p.7)

*“Next to me was an Albanian woman giving birth, she called the nurse over and gave her a gold bracelet, then the nurse and doctor were the whole time next to her, but they hardly looked at me.” (*[58], p.7)

### Making things better

Three Romani women described how they overcame poor experiences or barriers to accessing care; having a connection with the health centre [58], knowledge of rights [59], and the presence of Romani health workers [58].

One woman described that having a family member who was a health worker improved the doctors’ behavior, the quality of treatment received and the waiting time [58];

*“But then he saw that my mother is a health worker, his behavior changed and he apologized.” (*[58], p.6)

Another woman explained that her knowledge of her entitlement to care enabled her to overcome an unfair request for additional payment [59];

*“I started to go for regular check-ups with a [gynaecologist] who at first sought money from me, but, after telling him that I know that if a woman is pregnant, she shouldn’t pay for the checkups and that I could sue, I didn’t pay for anything […] I didn’t pay the 200 Macedonian denar [equivalent to 4 US dollars] because I knew I don’t have to…” (*[59], p.529)

Another woman described that the quality of care she received was improved as a Romani obstetrician intervened in her treatment to prevent an unnecessary Caesarean Section [58]. By contrast another woman reported that the verbal abuse she experienced was perpetrated by all the staff caring for her including a Romani nurse [59].

#### Line of argument synthesis

*Romani women in Europe report that they experience various forms of mistreatment within maternity care and barriers to accessing maternity care. Some of these experiences are discriminatory on the basis of multiple grounds including Roma ethnicity, economic situation, place of residence, and language. Awareness of rights to care and a relational connection with health service staff they encounter may reduce ‘othering’, and increase Roma women’s agency to improve the care they receive.*

Given the few studies included, the medium and low CERQual scores for these findings and the lack of quantitative data for comparison, this line of argument synthesis is tentative. When describing their experiences, the Romani women did not always make a comparison with the experience of other women, or propose a reason for the mistreatment or barriers they experienced. Only one of the included studies considered Romani women’s understanding of the concept of discrimination [58]. The researchers reported that there was often little understanding of this concept particularly amongst the women with little education. Despite this, nearly all the respondents believed that the treatment of Romani women was better in Western Europe, and there was implicit evidence in the data that some Romani women felt the mistreatment they experienced was on the grounds of their ethnicity.

Data from health workers were limited and were only presented in one included study [58], which included eight gynaecologists in Serbia and Macedonia. The respondents described the Romani women as being mostly uneducated, having lower literacy levels and health knowledge, being non-compliant, not listening, having large numbers of children and having an inherent “gypsy fear”. Negative attitudes were mostly directed towards the most uneducated Romani women.

In order to test and refine the tentative line of argument, a comparison was undertaken with the grey literature.

#### Findings from the grey literature

The grey literature included qualitative accounts from women; qualitative accounts from staff; and one report of an intervention. In this section, the first set of data are compared with the synthesis above, to see how well the line of argument explains the broader data from women’s own experiences.

Given the paucity of data on staff views from the qualitative research data, the staff views in the grey literature are presented in more depth, as a basis for future more detailed research. The single intervention is described in some detail, again, as the basis for future intervention studies in this area.

### Testing the line of argument: women’s views and experiences

Six sources of grey literature contained qualitative data about Romani women’s experiences of maternity care [36, 61–66]. These revealed that childbearing Romani women experienced poorer care than non-Roma women on the basis of their ethnicity, poverty, and place of residence and confirms the line or argument synthesis. No reports highlighted any positive experiences of maternity care in Europe.

The Romani women reported they were not treated with respect [36], were abandoned by maternity care health professionals [61, 62], denied treatment [61, 63], attended by underqualified staff [61, 62], and subjected to verbal or physical abuse [61–64] and degrading or humiliating treatment [63].

*“Doctors speak to you like you would speak to a dog.”* ([62], p.9)

*“When I was in the delivery room…The doctor started to call me names (Gypsies) and hit me really hard on my face. The nurse who was attending me hit me on my legs. It hurt, it gave me bruises.” (*[63], p.83)

Women reported that they were obliged to wait until all the non-Roma patients had been seen before being attended to by maternity healthcare professionals [63, 64], they were not allowed to have family members present with them during labour and delivery [63], their newborns were sometimes detained in the medical facilities until they had paid for their treatment [62, 64, 65], medical treatment was undertaken without consent [61] and in the most extreme cases women had been subjected to forced or coerced sterilisation [61, 63].

*“They took me to the operation theatre the next day…Before I was released, they gave me something to sign, but I did not know what it was and they did not explain it to me. Later I was given a medical release report where it was written that I was sterilized.” (*[63], p.64)

There was widespread evidence of racial segregation of maternity wards in Central and Eastern European regions, with Romani women reporting that the “Gypsy rooms” are of poorer quality, not cleaned by the hospital staff, not heated and contain fewer facilities including toilets [61–65].

*“Gypsy room…It is like in a concentration camp there.” (*[63], p.75)

Five of the sources of grey literature revealed reports from Romani women about barriers to accessing care which clearly resonate with the findings in the published literature and confirm the line of argument synthesis [36, 61, 62, 64, 65]. Barriers included lack of information about the healthcare system [36], language barriers and lack of provision of translation services [36], and financial barriers including the requirement of informal payments or lack of access to documentation or insurance services that were prerequisites for care [36, 61, 62, 65].

*“If you have money – you will have a baby, if you don’t have money – you won’t have a baby.” (*[62], p.10)

The women also had to overcome poor local infrastructure, lack of public transport services and lack of willingness of emergency services to attend the Roma settlements in order to access care [61, 64]. Some women reported that they did not access care as they were fearful of the poor treatment they would experience, others avoided care as they were fearful of the possible involvement of social care services and the removal of their children [36].

### Attitudes of health professionals caring for Romani women

The line of argument appears to be robust in terms of women’s views and experiences. However, it does not have sufficient explanatory power as the basis for a potential future solution in the absence of substantial data from the staff who are accused of discriminatory behaviours. This section provides insight on this aspect from the grey literature.

Six studies included interviews with health professionals working with pregnant Romani women [36, 61–65]. Two of these included data concerning the attitudes of health professionals towards childbearing Romani women in Spain, Hungary, Bulgaria and Slovakia, which consisted of only negative and discriminatory views and no positive attitudes [61, 63].

These health professionals expressed racist remarks concerning excessive birth rates amongst the Roma, their abuse of the social welfare system, their unwillingness to find decent work and irresponsibility about their lives and the lives of their children [61, 63].

*“They start having babies at the age of 12. It is worthless to instruct them. They all know about contraceptives but they have babies on purpose. They know that they will have family allowance if they have children.” (*[61], p.64)

“*Gypsies make their living on irregular work, robbery and the usage of the elders’ pensions. Only 10% of them have a decent job. They expect a lot but do very little.”* ([61], p.65)

*“Roma are poor…parents encourage children to steal, and they teach them to hate white people.”* ([63], p.88)

One report found that medical professionals stated that they believed that Romani women to be promiscuous, that they leave the hospital early after delivery to return to their husbands to have sexual intercourse [63],

*“Romani women…have intercourse all the time, even while pregnant…have several partners, are promiscuous, travel a lot, and bring diseases with them from other countries.” (*[63], p.54)

*“Mothers frequently leave the hospital without their babies…because they have to go home to be available for their husbands…for sex.” (*[63], p.88)

The health professionals also described Romani women as irresponsible, neglectful of their health [63], less intelligent than non-Roma women [61, 63], trouble-makers, degenerate, less civilized and less human [63].

*“Roma are dull-witted. There is no point to explain to them anything because they will not understand anyway, and it is intellectually exhausting to deal with Romani patients.” (*[61], p.65)

*“Romani women give birth quite easily. More intelligent women give birth with more difficulty, it is something in the brain.”* ([63], p.87)

*“They neglect their health and health problems.”* ([63], p.74)

Some health professionals considered the women to be lacking maternal instincts, and that they intermarried to purposely conceive disabled children to increase their benefit allowance [61, 63].

*“Roma leave [the hospital] early because of insufficient maternal instincts. Even an animal doesn’t leave its baby.”* ([63], p.88)

Health care staff in five reports confirmed the practice of segregation of the maternity wards [60–64]. Within these reports staff denied it was discriminatory, and justified this practice on the basis that it was done for hygienic reasons [61], to spare the Romani women from the discriminatory attitudes of the other non-Roma women and their families [61, 62], that it was at the request of the Romani women who wanted to be other Romani women [61, 64, 65], was on account of the Romani women being smokers [60] and was necessary to respect the rights of the white non-Roma women [61–63]. Some said that they were powerless to transfer a Roma patient to a non-Roma ward as it was decided by higher authority in the institutions [64].

*“I’m very careful so Roma won’t feel discriminated against, but Romani women want to be separated.” (*[63], p.78)

*They [Roma women] all want to be together in one room, even if they had to share one bed in the Gypsy room.” (*[63], p.78)

*“White women do not want to be with primitive, uneducated Romani women.” (*[63], p.78)

Some health professionals also justified denial of emergency services to respond to calls from Romani women on the basis that they were misusing the services because they were free or because it was more comfortable and they didn’t have to wait for their appointment with doctors [61, 63].

*“Most Romani women are abusing ambulances by saying they don’t have a car when they do…They lie to bring the ambulance because then they are treated immediately in the hospital.”* ([63], p.81)

#### Amending the line of argument synthesis

On the basis of these data, the line of argument synthesis could be reframed as follows*:*

*Romani women in Europe report that they experience various forms of mistreatment within maternity care and barriers to accessing maternity care. Some of these experiences are discriminatory on the basis of multiple grounds including Roma ethnicity, economic situation, place of residence, and language. Maternity health care staff accounts indicate that they are believe the Roma to be criminal, unintelligent, abusers of the welfare and health system, and bad mothers. In these accounts, health professionals deny discriminatory treatment and provide justification for segregation of maternity wards and denial of emergency services. This underlying prejudice contributes to maternity health care for Romani women that fails to meet standards of availability, acceptability, accessibility and quality. Awareness of rights to care and a relational connection with health service staff encounter may reduce ‘othering’ for Romani women, and increase their agency to improve the care they receive.*

### Intervention to address maternity service provision to Romani women

Given the data on poor reproductive outcomes amongst Roma women, and the evidence of widespread discriminatory attitudes it is surprising that there have been no formal intervention studies designed to address these issues. The single report located by the search strategy of an intervention study in the grey literature is described in this section, and compared with the emerging line of argument synthesis above, to see how the underlying programme theory for the intervention might fit with the findings of this review, and how this could, potentially, trigger improved outcomes.

The study is the Roma Health Mediation (RHM) Programme in Romania [66]. This programme aimed to improve the health status of pregnant and postpartum Romani women, infants and children by implementing health mediators from the local community. The evaluation report does not include an analysis of the philosophy of the RHM programme, attitudes of staff or experiences of the women or mediators, or the underlying mechanisms that were hypothesised to result in an increase in access to services. However, several features can be identified which would suggest that the programme was designed to overcome discrimination in the system. This included explicit intentions for the Roma health mediators to provide basic health education, raise awareness of the right to free health insurance and assist Roma women to obtain it, promote improved communication between the Roma and healthcare practitioners, and to raise awareness of the need for antenatal care and right to access care.

The evaluation was a retrospective analysis of outcomes before and after implementation of the programme. The time period for the evaluation was 2002–2008, and the number of localities included during this time increased from 42 to 281. Post-implementation outcomes were separated into “up to 2 years after implementation” and “more than 2 years after implementation”. Data sources included the 2000–2008 Vital Statistics Natality (VSN) files and the Vital Statistics Mortality (VSM) files, as well as data from The Roma Health Mediators’ registry and the Roma Inclusion Barometer 2006.

The report found that the RHM programme resulted in improved uptake of antenatal care by Romani women, an increase in length of time breastfeeding, and a reduction in the local stillbirth rate and infant mortality rate. There was no apparent effect on other infant outcomes including low birthweight and preterm birth. Survey data generated alongside the intervention demonstrated that Roma in the localities where the RHM programme had been implemented for more than two years felt significantly less discrimination in general and even less discrimination in hospitals and medical facilities when compared to Roma in localities where the programme had not yet been implemented or had been implemented very recently.

## Discussion

### Limitations

Considering only studies published in English may have introduced language bias and excluded relevant studies published in other languages [41]. This is particularly relevant as the Romani community is located widely across Europe, where studies and reports may be published in local languages only. There were no published intervention studies or quantitative surveys identified. There were only a very small number of included published qualitative studies, focussed on a few settings. None of the included studies made any comparisons with non-Roma women, so the findings may be common to a wide range of groups that are discriminated against, and not just to Roma women in particular. There were limited data that considered the perspectives of health providers, and the CERQual assessment of the review findings resulted in medium and low confidence results for all the findings

The authors of the grey literature often did not specify the methodology that had been adopted, and quality assessment could not be undertaken. Many of the sources had been funded or commissioned by organisations keen to promote Romani women’s rights and expose experiences of poor care, and hence publication bias cannot be excluded. There may be further sources of grey literature that this search did not identify. An additional source of relevant grey literature [67] was highlighted to us by an expert in the field after we had completed the analysis. This report, however, served as a further confirmatory check on the findings from the review about Romani women’s’ experiences of maternity care in Europe.

### The experiences of Romani women in a global context

The experiences of childbearing Romani women can be contextualised by a wider body of global evidence concerning disrespect, abuse and mistreatment of childbearing women in healthcare facilities and poor maternity healthcare professional behaviours, which include; physical, sexual and verbal abuse, stigma and discrimination, lack of communication or information, neglect or abandonment of patients, refusal to deliver services, lack of informed consent, lack of willingness to accommodate traditional practices, breaches of confidentiality or privacy and detention in facilities [17, 18, 68]. The experience of mistreatment in maternity care is occurring across the world in low-middle- and high-income settings, and disadvantaged or marginalised women are particularly affected [18].

The recent Lancet Series on Maternal Health identifies that a global approach to equitable and quality maternal health is needed, through the implementation of respectful, evidence-based care for all childbearing women [69]. There has been growing international interest in the implementation of a model of maternity health care that addresses the mistreatment of childbearing women across the world, by promoting positive staff attitudes and behaviours [68]. To achieve this, it is crucial to employ context-specific solutions that address the underlying macro, meso and micro-level contributing factors. Globally these include; lack of regulation or legal framework for health rights, poor working conditions, heavy workloads, long working hours, shortages of equipment, cultural norms and provider beliefs [18, 68, 70]. Interventions have included the implementation of mechanisms to ensure accountability to professional standards and ethics at all levels of the health system and increasing patient knowledge of their rights to an acceptable standard of treatment by healthcare providers [68]. Strategies have also included advocacy measures, addressing laws, policies and local protocols, investment in health facilities and salaries of health workers and education and training of health workers, particularly related to interpersonal and communication skills [70].

### Anti-Gypsyism

Anti-Gypsyism, is a specific form of racism that is focussed on groups that are encompassed by the stigmatising term “Gypsy”, which includes Roma, Sinti, and Travellers [71]. In common with other forms of racism, it is undergirded by the construction of the “otherness” of the “othered” group, in this case “Gypsies”, who are considered to share certain negative characteristics, that make them inferior, and not worthy of equal treatment [71]. The dehumanisation of those considered to be in the outgroup involves the denial of uniquely human characteristics such as intellectual ability, agency and emotional responsiveness, and results in the justification of discrimination [72]. The “othering” and dehumanisation of childbearing Romani women was clearly demonstrated in this review by comments made by health professionals about their nature and intelligence, and the subsequent justification of discriminatory behaviours including the segregation of wards and denial of emergency services.

### Strength of the line of argument synthesis as the basis for a future intervention study

On the basis of the line of argument synthesis, it is hypothesised that addressing maternity health professionals underlying prejudice and “othering” of the Roma in Europe is crucial to reduce discriminatory mistreatment within maternity care and to improve Romani women’s experience of care and access to care. This hypothesis is in line with the European Commission against Racism and Intolerance recommendations to Council of Europe member states to combat anti-Gypsyism in healthcare, which includes to provide training to health workers aimed at combating stereotypes, prejudice and discrimination [73]. Whilst the Council of Europe does not specify the types of training health workers should receive to combat antigypsyism, there is a need for the development of interventions that go beyond traditional healthcare diversity training or cultural competency or cultural awareness training. These have been widely criticised for increasing stereotyping and reinforcing essentialist racial identities [74, 75], thus accentuating the “otherness” of cultural groups and failing to address personal bias or prejudice [76].

When considering the RHM programme, further analysis in other areas of health have suggested that it improves doctors’ cultural competency and understanding of the Roma community [77, 78] and that this then leads to doctors being less likely to engage in discriminatory behaviour including the use of abusive language [77]. In the context of the RHM programme as presented in this review, it is not clear whether incorporating a cultural competency component has impacted on its success positively or negatively, nor is it clear if cultural competency was a specific aim or a by-product of the programme. Further analysis of the impact of the other elements of the programme on the doctors’ views of the “otherness” of the Roma is warranted, including how the doctors’ contact with the Roma mediators impacts on their underlying prejudice.

The line of argument synthesis findings indicate that re-categorisation of Romani women by individual health professionals, for example as “intelligent” or “relative of a co-worker”, positively impacted on their experience in different maternity care settings. This is in line with social psychology theory of multiple categorisation to overcome dehumanisation, which suggests the simple categorisation of in-group and out-group that leads in to intergroup discrimination [79] can be overcome by the use of multiple criteria for social categorisation [72]. Here, the availability of multiple criteria means that judgements based on criteria are no longer meaningful [72] and results in de-categorisation, the cognitive reconstruction of the target as an individual rather than a member of an oppositional group [80], hence inhibiting pre-existing stereotypes [81]. Although there are not yet examples within healthcare settings, models that incorporate multiple categorisation techniques, whereby participants are instructed to think about the multiple affiliations that characterise a target “outgroup”, rather than single affiliations, have been demonstrated to reduce intergroup bias, stereotypes, prejudice, dehumanisation and linguistic discrimination [72, 81–86].

The development of interventions utilising multiple categorisation techniques could enable health professionals working with Romani women in Europe to overcome dehumanising stereotypes that were demonstrated in the line of argument synthesis, and hence improve the quality, availability, acceptability and accessibility of maternity services to these women. The development of such interventions to address underlying health professional beliefs and prejudice could be transferable both geographically and contextually, and may benefit childbearing women who are experiencing discrimination and poor experiences of maternity care on the basis of other prohibited grounds.

## Conclusion

This review has demonstrated that Romani women in Europe experience various forms of discriminatory mistreatment within maternity care and barriers to accessing maternity care. The testing of the line of argument synthesis against grey literature findings confirmed the key elements of the synthesis, but also suggested that where particular Romani women had characteristics that led health care providers to see them as individuals rather than as ‘other’, underlying prejudice and discrimination could be overcome. Multiple categorisation techniques could be a viable basis for future interventions in this group, and for other marginalised population groups.

## Additional files

Additional file 1: Search Strategy. (DOCX 13 kb)
Additional file 2: Excluded Research Studies. (DOCX 14 kb)

## Abbreviations

AMSTAR: Assessing the methodological quality of systematic reviews
CASP: Critical appraisal skills programme
CERQual: Confidence in the evidence from reviews of qualitative research
EU: European Union
UNFPA: United Nations Population Fund
