# Supplementary material for: Discrimination against childbearing Romani women in maternity care in Europe: a mixed-methods systematic review
Source: Reprod Health. 2017 Jan 5;14:1. doi: 10.1186/s12978-016-0263-4 (PMC5217576; doi:10.1186/s12978-016-0263-4)
Supplement: Additional file 1: — Search Strategy. (DOCX 13 kb) [file 12978_2016_263_MOESM1_ESM.docx]

**Search strategy**

**Ovid SP- Medline (R) In Process and other Non-Indexed Citations and Ovid Medline (R) 1946-present, EMBASE 1974 to 04 May 2015, Maternal and Infant Care 1971 to April 2015**

1. (matern$ or pregnant or pregnancy or mother* or reproduct$).ab,ti.
2. (prenatal or pre-natal or prepartum or pre-partum or antenatal or ante-natal or perinatal or postnatal or post-natal or postpartum or post-partum or pueperium or puerperal, or intrapartum or intranatal, or birth or parturition or childbearing or child-bearing or childbirth or breastfeed$).ab,ti.
3. (obstetrics or midwifery or midwife or midwives).ab,ti.
4. (Roma or rroma or romani or gyps$) ti, ab.
5. 1 or 2 or 3
6. 4 and 5

**EBSCOhost EJS - AMED, CINAHL, Academic Search Complete, PsychINFO and Wilson Social Science Abstracts**

S3 S1 and S2

S2 TI (Roma or rroma or romani or gyps*) OR AB (Roma or rroma or romani or gyps*)

S1 TI ( matern* or pregnant or pregnancy or mother* or reproduct* ) OR AB ( matern* or pregnant or pregnancy or mother* or reproduct* ) OR TI (prenatal or pre-natal or prepartum or pre-partum or antenatal or ante-natal or perinatal or postnatal or post-natal or postpartum or post-partum or pueperium or puerperal, or intrapartum or intranatal, or birth or parturition or childbearing or child-bearing or childbirth or breastfeed*) OR AB (prenatal or pre-natal or prepartum or pre-partum or antenatal or ante-natal or perinatal or postnatal or post-natal or postpartum or post-partum or pueperium or puerperal, or intrapartum or intranatal, or birth or parturition or childbearing or child-bearing or childbirth or breastfeed) OR TI ( obstetrics or midwifery or midwife or midwives ) OR AB ( obstetrics or midwifery or midwife or midwives)

**PROSPERO search (01/01/1950 to 04/05/2015)**

Rroma OR Roma OR Romani or Gyps* (any field)
