# Supplementary material for: Discrimination against childbearing Romani women in maternity care in Europe: a mixed-methods systematic review
Source: Reprod Health. 2017 Jan 5;14:1. doi: 10.1186/s12978-016-0263-4 (PMC5217576; doi:10.1186/s12978-016-0263-4)
Supplement: Additional file 2: — Excluded Research Studies. (DOCX 14 kb) [file 12978_2016_263_MOESM2_ESM.docx]

**Additional file 2**

**Excluded research studies**

| **Author and date** | **Reason for exclusion** |
| --- | --- |
| Kosa, Lenart & Adany (2002) | Language - Hungarian |
